# Retraction Note: LncMAPK6 drives MAPK6 expressionand liver TIC self-renewal

**DOI:** 10.1186/s13046-022-02517-9

**Published:** 2022-10-20

**Authors:** Guanqun Huang, Hui Jiang, Yueming He, Ye Lin, Wuzheng Xia, Yuanwei Luo, Min Liang, Boyun Shi, Xinke Zhou, Zhixiang Jian

**Affiliations:** 1 Department of general surgery, The Fifth Affiliated Hospital of Guangzhou Medical University, Guangzhou, Guangdong Sheng China; 2 Department of Abdominal Oncology, The Fifth Affiliated Hospital of Guangzhou Medical University, Guangzhou, Guangdong Sheng China; 3 Department of General Surgery, Guangdong General Hospital, Guangdong Academy of Medical Sciences, Guangzhou, Guangdong Sheng China

**Retraction Note: J Exp Clin Cancer Res 37, 105 (2018)**

               **https://doi.org/10.1186/s13046-018-0770-y**

The Editor-in-Chief has retracted this article at the corresponding author’s request. After publication, the authors became aware that the results of the study were not reproducible, which undermined the conclusions of the article. Additional checks by the Publisher revealed considerable similarities between this article and publications from other authors [1–6], including text overlap, study design, and similar figures.

The authors have been unable to retrieve the original data or ethics documents.

Zhixiang Jian does not agree to this retraction. Hui Jiang, Yueming He, Ye Lin, Wuzheng Xia, Yuanwei Luo, Min Liang, Boyun Shi and Xinke Zhou have not responded to any correspondence from the editor or publisher about this retraction. The Publisher has not been able to obtain a current email address for Guanqun Huang.
